# Supplementary material for: Identification of photoperiod-induced specific miRNAs in the adrenal glands of Sunite sheep (Ovis aries)
Source: Front Vet Sci. 2022 Jul 22;9:888207. doi: 10.3389/fvets.2022.888207 (PMC9354845; doi:10.3389/fvets.2022.888207)
Supplement: Supplementary file 1 [file Data_Sheet_1.ZIP › Supplementary file/miRDeep2 file.zip!/miRDeep2 file/Know_Structure/SP42A2/oar-mir-17.pdf]

miRBase precursor : oar-mir-17
Total read count : 4533
oar-miR-17-5p read count : 4458
remaining reads : 75

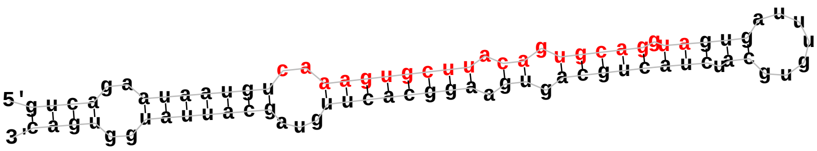

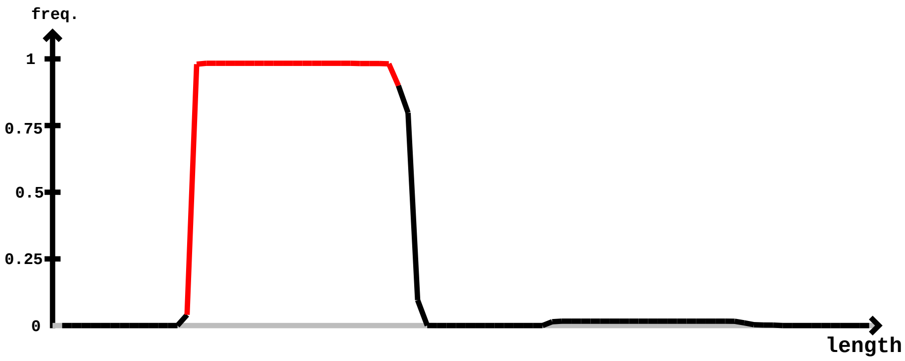

oar-miR-17-5p

```
5'- gucagaauaaugucaaagugcuuacagugcagguagugauuugugcaucuacugcagugaaggcacuuguagcauuauggugac   -3'    exp
    ((((..(((((((..((((((((.((.(((((.(((((.......)).))))))))).)).))))))))...)))))))..))))   reads      mm        sample
    ............ucaaagugcuuacagugc......................................................   1          0         seq
    ............ucaaagugcuuacagugcagg...................................................   2          0         seq
    ............ucaaagugcuuacagugcaggu..................................................   31         0         seq
    ............ucaaagugcuuacagugcaggua.................................................   50         0         seq
    ............ucaaagugcuuacagugcagguag................................................   98         0         seq
    ............ucaaagugcuuacagugcagguagu...............................................   1          0         seq
    .............caaagugcuuacagugU......................................................   1          1         seq
    .............caaagugcuuacagugc......................................................   2          0         seq
    .............caaagugcuuacagugcagg...................................................   3          0         seq
    .............caaagugcuuacagugcaggu..................................................   333        0         seq
    .............caaagugcuCacagugcaggu..................................................   7          1         seq
    .............caaagugcuCacagugcaggua.................................................   4          1         seq
    .............caaagugcuuacagugcaggua.................................................   404        0         seq
    .............caaagugcuCacagugcagguag................................................   51         1         seq
    .............caaagugcuuacagugcagguag................................................   3023       0         seq
    .............caaagugcuuacagugcagguagu...............................................   423        0         seq
    .............caaagugcuCacagugcagguagu...............................................   8          1         seq
    .............caaagugcuuacagugcagguagug..............................................   1          0         seq
    ..............aaagugcuuacagugcaggua.................................................   2          0         seq
    ..............aaagugcuuacagugcagguag................................................   12         0         seq
    ..............aaagugcuuaUagugcagguag................................................   1          1         seq
    ..................................................acugcagugaaggcacuug...............   2          0         seq
    ..................................................acugcagugaaggcacuugu..............   27         0         seq
    ..................................................acugcagugaaggcacuugua.............   31         0         seq
    ..................................................acugcagugaaggcacuuguag............   5          0         seq
    ...................................................cugcagugaaggcacuuguag............   1          0         seq
    ...................................................cugcagugaaggcacuuguagc...........   1          0         seq
    ...................................................cugcagugaaggcacuuguagca..........   8          0         seq
```
